# After the games are over: life‐history trade‐offs drive dispersal attenuation following range expansion

**DOI:** 10.1002/ece3.2314

**Published:** 2016-08-18

**Authors:** T. Alex Perkins, Carl Boettiger, Benjamin L. Phillips

**Affiliations:** ^1^ Department of Biological Sciences and Eck Institute for Global HealthUniversity of Notre DameNotre DameIndiana; ^2^ Department of Environmental Science, Policy, & ManagementUniversity of California, BerkeleyBerkeleyCalifornia; ^3^ School of BiosciencesUniversity of MelbourneMelbourneVic.Australia

**Keywords:** Fitness, life‐history evolution, natural selection, theory, traveling wave

## Abstract

Increased dispersal propensity often evolves on expanding range edges due to the Olympic Village effect, which involves the fastest and fittest finding themselves together in the same place at the same time, mating, and giving rise to like individuals. But what happens after the range's leading edge has passed and the games are over? Although empirical studies indicate that dispersal propensity attenuates following range expansion, hypotheses about the mechanisms driving this attenuation have not been clearly articulated or tested. Here, we used a simple model of the spatiotemporal dynamics of two phenotypes, one fast and the other slow, to propose that dispersal attenuation beyond preexpansion levels is only possible in the presence of trade‐offs between dispersal and life‐history traits. The Olympic Village effect ensures that fast dispersers preempt locations far from the range's previous limits. When trade‐offs are absent, this preemptive spatial advantage has a lasting impact, with highly dispersive individuals attaining equilibrium frequencies that are strictly higher than their introduction frequencies. When trade‐offs are present, dispersal propensity decays rapidly at all locations. Our model's results about the postcolonization trajectory of dispersal evolution are clear and, in principle, should be observable in field studies. We conclude that empirical observations of postcolonization dispersal attenuation offer a novel way to detect the existence of otherwise elusive trade‐offs between dispersal and life‐history traits.

## Introduction

When a population spreads across space, several evolutionary forces come into play that should drive the evolution of increased dispersal propensity as the invasion front moves forward (Travis and Dytham 2002). First, under the Olympic Village effect (Phillips et al. 2008), inhabitants at the farthest reaches of the invasion front tend to be limited to the most capable dispersers (Shine et al. 2011; Benichou et al. 2012), leading to spatially assortative mating by dispersal propensity and the perpetuation of this effect in subsequent generations (Phillips et al. 2010). More recently, this phenomenon has been referred to as “spatial sorting” (Shine et al. 2011), which we adopt henceforth. Second, in a density‐regulated context, these highly dispersive phenotypes arriving on the invasion front benefit from a fitness advantage through lowered competition with conspecifics (Phillips et al. 2008). Finally, this fitness advantage of more dispersive types may increase over time, as life‐history traits also undergo adaptive evolution in the vanguard population (Perkins et al. 2013).

Despite the fact that these distinct evolutionary forces have only recently been elucidated, there is a rapidly growing body of empirical work showing that dispersal propensity often does increase on invasion fronts. Spreading populations ranging from trees to ants, crickets, beetles, and amphibians have all shown evidence of such increases as their ranges have expanded (Cwynar and MacDonald 1987; Simmons and Thomas 2004; Alford et al. 2009; Leotard et al. 2009; Lombaert et al. 2014). These rapid increases in dispersal propensity have broad implications for ecological management (e.g., the management of invasive species, or native species shifting under climate change) and even medicine (van Ditmarsch et al. 2013; Orlando et al. 2013). Although there is growing appreciation for the evolution of increased dispersal propensity in the low‐density environments along expanding range edges, much less is known about what happens after the range edge has passed and equilibrium densities have been attained.

The evolutionary trajectory of dispersal after colonization is important to understand for at least three key reasons. First, it gives an indication of the long‐term consequences of invasion on evolution. If the evolution of increased dispersal is a transient phenomenon, with reversion to preinvasion levels following colonization, then its long‐term implications are modest. If, however, dispersal tends to be maintained at high levels following establishment, then a persistent cline in dispersal phenotypes is expected to exist across the species' range, with implications for population dynamics and life‐history evolution. In this case, invasion may also be a driver of diversification, with many instances of geographic variation being a product of past invasions (Phillips et al. 2010) rather than local adaptation along an underlying environmental gradient (Kirkpatrick and Barton 1997). Second, understanding the postcolonization trajectory of dispersal could potentially yield further insight into evolutionary processes occurring on the invasion front. Trade‐offs between dispersal and fitness, for example, may alter the evolutionary and spread dynamics of the invasion front (Burton et al. 2010; Orlando et al. 2013). If such a trade‐off exists, it may manifest as a rapid attenuation of dispersal propensity behind the invasion front. Third, and related to the first point, the reality is that the vast majority of well‐documented examples of spread pertain to events that have more or less concluded (Perkins 2012). Documenting the spread of an invasive species takes time – time in which the population is filling its new range – and so invasions are often only well documented as they are reaching their conclusion. Because of this, inferences about evolutionary processes on invasion fronts will often be made by examining populations at numerous times postcolonization, a kind of space‐for‐time substitution (Phillips et al. 2008). Such inferences depend critically on knowing the extent to which populations sampled postcolonization resemble populations on the invasion front when it originally passed that location.

Here, we attempt to provide clarity about the processes that govern the evolution of dispersal from the moment of colonization onward. It is clear from a limited number of theoretical and empirical studies that populations may evolve attenuated dispersal propensity following colonization (Duckworth and Badyaev 2007; Burton et al. 2010; Lindstrom et al. 2013). One prominent example in bluebirds attributed postcolonization dispersal attenuation to trade‐offs between dispersal and life‐history traits (Duckworth and Badyaev 2007). This is a straightforward invocation of natural selection – less dispersive individuals, who also happen to be less aggressive, invest more in parental care and so increase in frequency over time. Another recent example in cane toads attributed postcolonization dispersal attenuation to spatial sorting (Lindstrom et al. 2013). This mechanism, whereby dispersal phenotypes are sorted along the strong density cline on the invasion front (Shine et al. 2011; Benichou et al. 2012), posits that the constantly shifting density cline creates a situation whereby the flow of dispersing individuals is asymmetric at any point along the cline. The idea is that this then results in less dispersive individuals from high‐density areas outnumbering more dispersive individuals from low‐density areas. Although the authors of previous studies of postcolonization dispersal attenuation likely had good reason to make one inference or another, they left the question of the generality of these mechanisms unresolved. Below, we develop a simple model and use it to determine the conditions under which these two mechanisms might operate following colonization. This theoretical analysis not only clarifies the likely importance of these mechanisms, but also provides suggestions about empirical signatures of trade‐offs between dispersal and life‐history traits that manifest in spatially extended populations.

## Methods

We performed a theoretical analysis comparing spatiotemporal patterns arising from a suite of nested dynamical models with differing assumptions about spatial sorting and fitness trade‐offs. Because our primary objective was to obtain a general understanding of which of a limited set of alternative assumptions might be most likely to generate postcolonization dispersal attenuation, we used a simple deterministic modeling framework with movement represented by a diffusion approximation (Okubo and Levin 2001). Although demographic stochasticity and genetic drift can be important for capturing the dynamics of small populations on an expanding range edge (Alleaume et al. 2006; Bridle et al. 2010), we opted to use a deterministic model in our analysis because of the clarity it provides about the relationships between key parameters of relevance to our objectives. Such use of deterministic models to perform initial scoping of major effects and stochastic models to perform more specific refinements is consistent with the development of theory about other aspects of the ecological and evolutionary dynamics of range expansion (Sexton et al. 2009).

### General ecological model

We consider a species with two phenotypes: slow dispersers with density *N*_s_(*t*, *x*) at time *t* and location *x*, and fast dispersers with density *N*_f_(*t*, *x*) . For convenience, we drop the notation indicating the specification of these variables over time and space. We assume that each type disperses according to a diffusion process with mean squared displacement per time *D*_s_ and *D*_f_, respectively. This serves as a deterministic approximation of average behavior of what is ultimately a stochastic process of individual movement in nature. At any particular location *x* in the absence of immigration and emigration, we model the dynamics of the two types using a Lotka–Volterra competition model with logistic population growth. One departure from this model that we make is that we decompose the intrinsic growth rates, *r*_s_ and *r*_f_, as *r*_s_ = (*ρ*_s_ + *b*_s_) − (*ρ*_s_ − *d*_s_) and *r*_f_ = (*ρ*_f_ + *b*_f_) − (*ρ*_f_ − *d*_f_). We interpret *ρ*_s_ and *ρ*_f_ as birth and death rates of each type when either is at its nonzero equilibrium, *b*_s_ and *b*_f_ as boosts in birth rates at low density, and *d*_s_ and *d*_f_ as reductions in death rates at low density (defined on [0, *ρ*_s_] and [0, *ρ*_f_], respectively). Together, these assumptions combine to yield

(1a) $$\begin{array}{cc} & \frac{\partial N_{s}}{\partial t}=D_{s}\frac{\partial^{2}N_{s}}{\partial x^{2}}+\rho_{s}N_{s}-\rho_{s}N_{s}+\left(\left(\rho_{s}+b_{s}\right)-\left(\rho_{s}-d_{s}\right)\right)\\ & \;\;N_{s}\left(1-a_{\mathrm{ss}}N_{s}-a_{\mathrm{fs}}N_{f}\right) \end{array}$$

(1b) $$\begin{array}{cc} & \frac{\partial N_{f}}{\partial t}=D_{f}\frac{\partial^{2}N_{f}}{\partial x^{2}}+\rho_{f}N_{f}-\rho_{f}N_{f}+\left(\left(\rho_{f}+b_{f}\right)-\left(\rho_{f}-d_{f}\right)\right)\\ & \;\;N_{f}\left(1-a_{\mathrm{ff}}N_{f}-a_{\mathrm{sf}}N_{s}\right). \end{array}$$

As specified, allowing for nonzero values of *ρ*_s_ and *ρ*_f_ is inconsequential to the model's dynamics, but we include them because allowing for turnover within the population at equilibrium is necessary for the model to capture evolutionary change at high density. We now consider one special case and one elaboration of this model that differ in their assumptions about the genetics of the fast and slow phenotypes.

### Genetic models

#### Case 1: Complete heritability

The simplest assumption that can be made about the genetics of these two types is that parents always beget like offspring. One example of when this might be a reasonable approximation would be for a clonal species with a single gene differentiating fast and slow types and infrequent mutation from one type to another relative to the timescale of spatial expansion. When this assumption holds, there is no need to consider nonzero *ρ*_s_ or *ρ*_f_, and we can collapse the birth and death rates down to their sums in *r*_s_ and *r*_s_. Next, to reduce the number of parameters and to clarify the scales of interest for each variable, we nondimensionalize the model [see Petersen and Hastings (2001) for an overview of this technique in ecology]. To do so, we first separate the dimensional and nondimensional components of each variable as $N_{s}=n_{s}N_{s}^{*}$, $N_{f}=n_{f}N_{f}^{*}$, *t* = $\tau t^{*}$, and *x* = $\chi x^{*}$, and then define their dimensional components as $N_{s}^{*}=a_{\mathrm{ss}}^{-1}$, $N_{f}^{*}=a_{\mathrm{ss}}^{-1}$, $t^{*}=r_{s}^{-1}$, and $x^{*}=\sqrt{D_{s}/r_{s}}$, to obtain

(2a) $$\frac{\partial n_{s}}{\partial \tau }=\frac{\partial^{2}n_{s}}{\partial \chi^{2}}+n_{s}\left(1-n_{s}-\alpha_{\mathrm{fs}}n_{f}\right)$$

(2b) $$\frac{\partial n_{f}}{\partial \tau }=D\frac{\partial^{2}n_{f}}{\partial \chi^{2}}+rn_{f}\left(1-\alpha_{\mathrm{ff}}n_{f}-\alpha_{\mathrm{sf}}n_{s}\right),$$

where *D* = *D*_f_/*D*_s_, *r* = *r*_f_/*r*_s_, *α*_fs_ = *a*_fs_/*a*_ss_, *α*_ff_ = *a*_ff_/*a*_ss_, and *α*_sf_ = *a*_sf_/*a*_ss_.

#### Case 2: Single locus in a sexual, diploid species

To consider the possibility that not all offspring will resemble their parental phenotypes, we extend the model to allow for the phenotype to be determined by a pair of alleles at a single locus. We assume that there are only two alleles segregating at this locus, with one resulting in the slow phenotype in individuals homozygous for that allele (the frequency of which is *p*) and the other resulting in the fast phenotype in individuals homozygous for that allele (the frequency of which is *q*). To allow for the full range of possibilities about the dominance of the fast and slow phenotypes, we assume that each heterozygote acquires the slow phenotype with probability *h*. Consequently, there are four states that we must follow: densities of each of the homozygotes, *N*_s,pp_ and *N*_f,qq_, and of heterozygotes with either of the phenotypes, *N*_s,pq_ and *N*_f,pq_. We assume that differences in birth rates are solely attributable to differences in female fecundity, that mating is random, and that inheritance follows Mendelian proportions. Because we also allow for the possibility of differences in death rates by offspring phenotype, our model's assumptions are incompatible with those of Hardy–Weinberg equilibrium. We therefore derive an explicit description of the dynamics of coupled demographic and population genetic change, which allows for the separate examination of each of the ways that a trade‐off could manifest in a system with Lotka–Volterra competition dynamics between two interbreeding phenotypes.

To follow the dynamics of the four states of interest consistent with these assumptions, we expand on equations (1a) and (1b) by taking the following steps: (1) separating *N*_s_ into *N*_s,pp_ and *N*_s,pq_ and *N*_f_ into *N*_f,pq_ and *N*_f,qq_; (2) adding terms for births of each type owing to matings between parents of all combinations of types in proportions delineated in Table 1; (3) separating dimensional and nondimensional components of each variable; (4) defining dimensional components as $N_{s}^{*}=a_{\mathrm{ss}}^{-1}$, $N_{f}^{*}=a_{\mathrm{ss}}^{-1}$, $t^{*}=r_{s}^{-1}$, and $x^{*}=\sqrt{D_{s}/r_{s}}$, where *r*_s_ = *b*_s_ + *d*_s_; and (5) performing the necessary algebra to obtain nondimensional equations

(3) $$\begin{array}{cc} \frac{\partial n_{s,\text{pp}}}{\partial \tau } & =\;\frac{\partial^{2}n_{s,\text{pp}}}{\partial \chi^{2}}+\left(\rho_{s}+\beta_{s}\left(1-n_{s}-\alpha_{\text{fs}}n_{f}\right)\right)\left({\vec{m}}_{s,s,\text{pp}}\cdot \left({\vec{n}}_{D,s}{\vec{\eta }}_{S,\cdot }\right)\right)\\ & +\left(\rho_{f}+\beta_{f}\left(1-\alpha_{\text{ff}}n_{f}-\alpha_{\text{sf}}n_{s}\right)\right)\left({\vec{m}}_{f,s,\text{pp}}\cdot \left({\vec{n}}_{D,f}{\vec{\eta }}_{S,\cdot }\right)\right)\\ & -\left(\rho_{s}-\delta_{s}\left(1-n_{s}-\alpha_{\text{fs}}n_{f}\right)\right)n_{s,\text{pp}} \end{array}$$

(4) $$\begin{array}{cc} \frac{\partial n_{s,\text{pq}}}{\partial \tau } & =\frac{\partial^{2}n_{s,\text{pq}}}{\partial \chi^{2}}+h\left(\rho_{s}+\beta_{s}\left(1-n_{s}-\alpha_{\text{fs}}n_{f}\right)\right)\left({\vec{m}}_{s,s,\text{pq}}\cdot \left({\vec{n}}_{D,s}{\vec{\eta }}_{S,\cdot }\right)\right)\\ & +\;h\left(\rho_{f}+\beta_{f}\left(1-\alpha_{\text{ff}}n_{f}-\alpha_{\text{sf}}n_{s}\right)\right)\left({\vec{m}}_{f,s,\text{pq}}\cdot \left({\vec{n}}_{D,f}{\vec{\eta }}_{S,\cdot }\right)\right)\\ & -\left(\rho_{s}-\delta_{s}\left(1-n_{s}-\alpha_{\text{fs}}n_{f}\right)\right)n_{s,\text{pq}} \end{array}$$

(5) $$\begin{array}{c} \begin{array}{c} \frac{\partial n_{f,\text{pq}}}{\partial \tau }=D\frac{\partial^{2}n_{f,\text{pq}}}{\partial \chi^{2}}+\left(1-h\right)\left(\rho_{s}+\beta_{s}\left(1-n_{s}-\alpha_{\text{fs}}n_{f}\right)\right)\\ \left({\vec{m}}_{s,f,\text{pq}}\cdot \left({\vec{n}}_{D,s}{\vec{\eta }}_{S,\cdot }\right)\right)+\left(1-h\right)\left(\rho_{f}+\beta_{f}\left(1-\alpha_{\text{ff}}n_{f}-\alpha_{\text{sf}}n_{s}\right)\right)\\ \left({\vec{m}}_{f,f,\text{pq}}\cdot \left({\vec{n}}_{D,f}{\vec{\eta }}_{S,\cdot }\right)\right)-\left(\rho_{f}-\delta_{f}\left(1-\alpha_{\text{ff}}n_{f}-\alpha_{\text{sf}}n_{s}\right)\right)n_{f,\text{pq}} \end{array} \end{array}$$

(6) $$\begin{array}{cc} \frac{\partial n_{f,\text{qq}}}{\partial \tau } & =\;D\frac{\partial^{2}n_{f,\text{qq}}}{\partial \chi^{2}}+\left(\rho_{s}+\beta_{s}\left(1-n_{s}-\alpha_{\text{fs}}n_{f}\right)\right)\left({\vec{m}}_{s,f,\text{qq}}\cdot \left({\vec{n}}_{D,s}{\vec{\eta }}_{S,\cdot }\right)\right)\\ & +\left(\rho_{f}+\beta_{f}\left(1-\alpha_{\text{ff}}n_{f}-\alpha_{\text{sf}}n_{s}\right)\right)\left({\vec{m}}_{f,f,\text{qq}}\cdot \left({\vec{n}}_{D,f}{\vec{\eta }}_{S,\cdot }\right)\right)\\ & -\left(\rho_{f}-\delta_{f}\left(1-\alpha_{\text{ff}}n_{f}-\alpha_{\text{sf}}n_{s}\right)\right)n_{f,\text{qq}}, \end{array}$$

where *ρ*_s_ = *ρ*_s_/*r*_s_, *ρ*_f_ = *ρ*_f_/*r*_s_, *β*_s_ = *b*_s_/*r*_s_, *β*_f_ = *b*_f_/*r*_s_, *δ*_f_ = *d*_f_/*r*_s_, *α*_fs_ = *a*_fs_/*a*_ss_, *α*_sf_ = *a*_sf_/*a*_ss_, and *α*_ff_ = *a*_ff_/*a*_ss_, and *η*_s,pp_, *η*_s,pq_, *η*_f,pq_, and *η*_f,qq_ are normalized frequencies of each type. Vectors of the densities of females of each phenotype–genotype combination (${\vec{n}}_{D,\cdot }$), the frequencies of males of each such combination (${\vec{n}}_{S,\cdot }$), and the proportion of each type of mating resulting in offspring of a given combination (${\vec{m}}_{\cdot ,\cdot ,\cdot }$) are provided in Table 1. Note the significance of the parameters *ρ*_s_ and *ρ*_f_, which allow for continued population turnover and evolution even when *r*_s_ = *r*_f_ = 0 and the population has ceased growing.

**Table 1:** Proportions of matings that result in offspring of a given phenotype and genotype

Dam	Sire	Offspring				Dam	Sire	Offspring			
		s,pp	s,pq	f,pq	f,qq			s,pp	s,pq	f,pq	f,qq
${\vec{n}}_{D,s}$	${\vec{\eta }}_{S,\cdot }$	${\vec{m}}_{s,s,\mathrm{pp}}$	${\vec{m}}_{s,s,\mathrm{pq}}$	${\vec{m}}_{s,f,\mathrm{pq}}$	${\vec{m}}_{s,f,\mathrm{qq}}$	${\vec{n}}_{D,s}$	${\vec{\eta }}_{S,\cdot }$	${\vec{m}}_{s,s,\mathrm{pp}}$	${\vec{m}}_{s,s,\mathrm{pq}}$	${\vec{m}}_{s,f,\mathrm{pq}}$	${\vec{m}}_{s,f,\mathrm{pq}}$
*n*_s,pp_	*n*_s,pp_	1	0	0	0	*n*_f,pq_	*n*_s,pp_	1/2	1/2*h*	(1/2)(1 − *h*)	0
*n*_s,pp_	*n*_s,pq_	1/2	(1/2)*h*	(1/2)(1 − *h*)	0	*n*_f,pq_	*n*_s,pq_	1/4	(1/2)*h*	(1/2)(1 − *h*)	1/4
*n*_s,pp_	*n*_f,pq_	1/2	(1/2)*h*	(1/2)(1 − *h*)	0	*n*_f,pq_	*n*_f,pq_	1/4	(1/2)*h*	(1/2)(1 − *h*)	1/4
*n*_s,pp_	*n*_f,qq_	0	*h*	1 − *h*	0	*n*_f,pq_	*n*_f,qq_	0	(1/2)*h*	(1/2)(1 − *h*)	1/2
*n*_s,pq_	*n*_s,pp_	1/2	(1/2)*h*	(1/2)(1 − *h*)	0	*n*_f,qq_	*n*_s,pp_	0	*h*	1 − *h*	0
*n*_s,pq_	*n*_s,pq_	1/4	(1/2)*h*	(1/2)(1 − *h*)	1/4	*n*_f,qq_	*n*_s,pq_	0	(1/2)*h*	(1/2)(1 − *h*)	1/2
*n*_s,pq_	*n*_f,pq_	1/4	(1/2)*h*	(1/2)(1 − *h*)	1/4	*n*_f,qq_	*n*_f,pq_	0	(1/2)*h*	(1/2)(1 − *h*)	1/2
*n*_s,pq_	*n*_f,qq_	0	(1/2)*h*	(1/2)(1 − *h*)	1/2	*n*_f,qq_	*n*_f,qq_	0	0	0	1

### Model analyses

Our primary interest was understanding patterns of the relative frequencies of the fast and slow types across space long after initial colonization. This required first determining the range of patterns that are possible and then assessing how different ecological scenarios affect those patterns. Because our interests were general and not in reference to any particular system, we limited our analyses to the nondimensionalized equations, which emphasize relative differences between the two types.

We solved each of the models under scenarios in which there was either no trade‐off (i.e., all life‐history parameters equal for both types) or a trade‐off in any of four life‐history parameters (i.e., differences in birth, death, or competition resulting in lower population growth of the fast type), implemented one at a time with the following values: *r *=* *0.8 (or *β*_f_ = *δ*_f_ = 0.4), *α*_ff_ = 1.1, *α*_fs_ = 0.9, and *α*_sf_ = 1.1. It is not clear a priori whether certain types of trade‐offs should impact patterns of postcolonization dispersal evolution in different ways, and so we considered all possible trade‐offs under our simple ecological model. Unless specified otherwise, *D *=* *1.2 was used as a default relative difference in dispersal, meaning that fast individuals have a 20% larger dispersal coefficient than slow ones. Whenever a life‐history trade‐off was not being implemented, default values of life‐history parameters were *r *=* *1, *β*_s_ = *β*_f_ = *δ*_s_ = *δ*_f_ = 0.5, *α*_fs_ = *α*_sf_ = *α*_ff_ = 1, and *ρ*_s_ = *ρ*_f_ = 1 or 0. In addition, we analyzed each scenario about life‐history trade‐offs under different assumptions about density dependence. For the model in equations (2a) and (2b), the possibilities were either density‐independent or density‐dependent growth. For the model in equations (3), (4), (5), (6), the possibilities included either density‐independent growth or density‐dependent growth with *ρ*_s_ = *ρ*_f_ = 0 or 1. We limited analyses of the latter model to *h *=* *0.5.

We solved the model in equations (2a) and (2b) on a space–time domain of [0, 40]_*χ*_ × [0, 100]_*τ*_ for density‐independent growth and [0, 40]_*χ*_ × [0, 400]_*τ*_ for density‐dependent growth, both under initial conditions of *n*_s_ = *n*_f_ = 0.1 at *χ* = 0 and *n*_s_ = *n*_f_ = 0 elsewhere. Conditions for the model in equations (3), (4), (5), (6) were the same but with the additional specification that all *η* = 0.25 at *τ* = 0, which is consistent with *n*_s_ = *n*_f_ and *h *=* *0.5 in a population at Hardy–Weinberg equilibrium. All solutions of the models were obtained numerically using the deSolve package (deSolve) in R (Soetaert et al. 2010; R Core Team 2010).

## Results

### Aspatial model: equilibrium properties

Before we examine dynamics under the spatial models, we first note the equilibrium properties of the nondimensionalized ecological model in a population with no emigration or immigration, as this is useful background information for interpreting the spatial results. Under default parameter values in which there are no life‐history trade‐offs, there are infinitely many unstable equilibria (${\hat{n}}_{s},{\hat{n}}_{f}$) satisfying ${\hat{n}}_{s}+{\hat{n}}_{f}=1$. In the more general case where some or all *α* ≠ 1, the equilibrium values of the two types in an isolated local population are either $\left({\hat{n}}_{s},{\hat{n}}_{f}\right)=\left(1,0\right),\;\left({\hat{n}}_{s},{\hat{n}}_{f}\right)=\left(0,\alpha_{\mathrm{ff}}^{-1}\right)$, or, in cases where the parameter values yield positive values for both types,

(7) $$\left({\hat{n}}_{s},{\hat{n}}_{f}\right)=\left(\frac{\alpha_{\mathrm{ff}}-\alpha_{\mathrm{fs}}}{\alpha_{\mathrm{ff}}-\alpha_{\mathrm{sf}}\alpha_{\mathrm{fs}}},\frac{1-\alpha_{\mathrm{sf}}}{\alpha_{\mathrm{ff}}-\alpha_{\mathrm{sf}}\alpha_{\mathrm{fs}}}\right).$$

Under this latter case, in which some or all *α* ≠ 1, the equilibrium and stability properties of the model are equivalent to those of the Lotka–Volterra model. The special case in which all *α* = 1 is not one that has been emphasized in studies of interspecific competition, but it is highly appropriate for examining the dynamics of two or more types of a single species with similar, but potentially differing, life‐history properties.

### Spatial model: density‐independent growth

**Figure 1:**
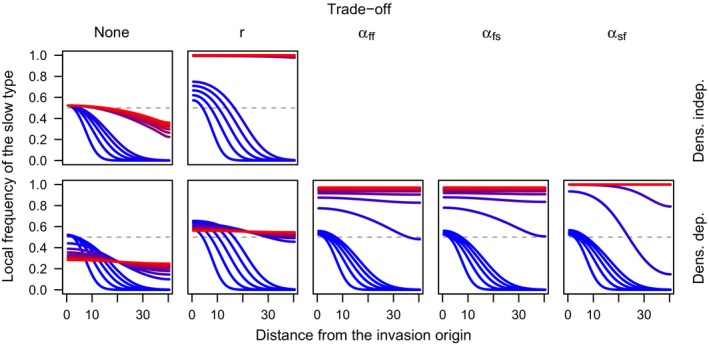
Spatial spread of the slow type under different scenarios about density dependence and life‐history trade‐offs under the clonal model in equations (2a) and (2b). Each curve shows the invasion profile of the slow type at a given point in time, with time indicated by colors ranging from blue at time *τ* = 0 to red at time *τ* = 100 in the top row and *τ* = 400 in the bottom row.

**Figure 2:**
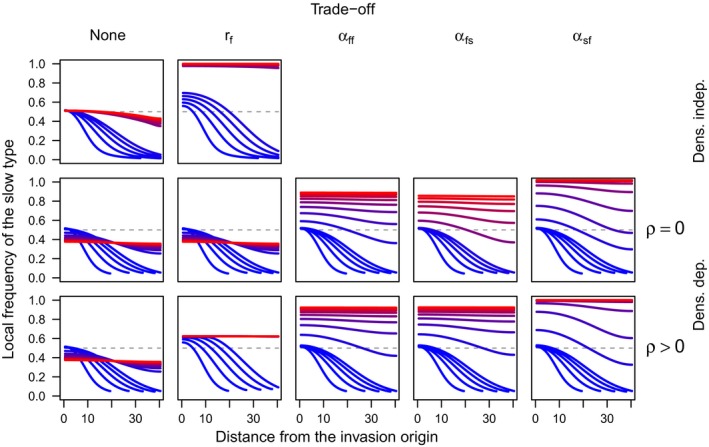
Spatial spread of the slow type under different scenarios about density dependence and life‐history trade‐offs under the diploid model in equations (3), (4), (5), (6). Each curve shows the invasion profile of the slow type at a given point in time, with time indicated by colors ranging from blue at time *τ* = 0 to red at time *τ* = 100 in the top row and *τ* = 400 in the bottom two rows.

Under either model with density‐independent population growth, the frequency of the slow type always approaches an equilibrium that is constant across the entire spatial domain (Figs. 1, 2). When the intrinsic growth rates of the two types are equal (indicated by the ratio *r *=* *1), the initial frequencies are approached across the entire domain as time *τ* → ∞ (Figs. 1, 2, top left). In the density‐independent case, there is a persistent density gradient present, even as *τ* → ∞. This density gradient sets up conditions for a net flux of genotypes from high‐density to low‐density areas; thus, we see the slow type invade and the long‐term frequencies slowly approach the initial frequencies across the entire domain.

### Spatial model: density‐dependent growth

**Figure 3:**
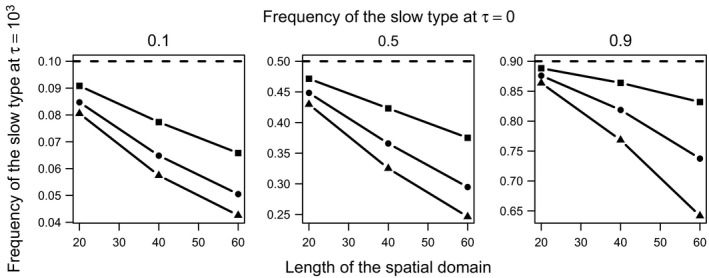
Frequency of the slow type across the entire spatial domain long after all locations have been invaded and equilibrium densities at each location have been attained (i.e., *τ* = 10^3^). These frequencies, which correspond to the frequency indicated by the red line in the bottom left panel of Figure 2, are shown here as a function of the length of the spatial domain (*x*‐axis), the initial frequency of the slow type (dashed lines, separate panels), and the relative dispersal ability of the fast type (square: *D *=* *1.1, circle: *D *=* *1.2, triangle: *D *=* *1.3).

The frequency of the slow type always approaches a constant equilibrium under models with density dependence, as well (Figs. 1, 2). In this case, when the intrinsic population growth rates of the two types are equal (i.e., *β*_s_ = *β*_f_ = *δ*_s_ = *δ*_f_ = 0.5), the equilibrium frequency of the slow type is always less than its frequency at introduction (Figs. 1, 2, bottom left). The reason that this equilibrium frequency is always less than the introduction frequency, unlike under the density‐independent model, is that growth ceases once equilibrium densities are attained. Thus, any numerical advantages that the fast type enjoys at locations far from the invasion origin are preserved in the long term, because any $\left({\hat{n}}_{s},{\hat{n}}_{f}\right)$ satisfying ${\hat{n}}_{s},{\hat{n}}_{f}=1$ is an equilibrium. However, because those equilibria are unstable, they shift in response to perturbations from dispersal from nearby locations with slightly different frequencies, eventually resulting in a homogenization of frequencies across space. The value of this spatially homogenized equilibrium frequency of the slow type depends on the initial frequency of the slow type, the relative dispersal advantage of the fast type, and the extent of the spatial domain (Fig. 3). With a greater relative dispersal ability and a larger spatial domain, the fast type will enjoy preemption of a greater area for a longer time, resulting in a lower equilibrium frequency of the slow type (Fig. 3).

Under all scenarios that we examined, life‐history trade‐offs always resulted in an increase in the equilibrium frequency of the slow type relative to what it was in the absence of the trade‐off (Figs. 1, 2). For a trade‐off in the intrinsic growth rate (indicated by the ratio of growth rates of fast and slow types *r *<* *1), the boost in the equilibrium frequency of the slow type attributable to this trade‐off is modest, because the trade‐off ceases to operate once equilibrium densities are attained (Fig. 1 and 2, second columns, bottom rows). Much like the intermediate equilibrium frequency of the slow type in the absence of trade‐offs (Fig. 3), the value of this equilibrium frequency is likely subject to the initial frequency of the slow type, the relative dispersal ability of the fast type, and the extent of the spatial domain, as well as the strength of the trade‐off. Other trade‐offs that we examined had much clearer effects, leading to eventual fixation of the slow type (Figs. 1, 2, three rightmost columns). Unlike a trade‐off in the intrinsic growth rate, the effect of trade‐offs in three different forms of relative competitive ability persisted after overall population growth slowed. The long‐term outcome of fixation of the slow type is therefore inevitable based on the equilibrium properties of the competition model given values of the interaction coefficients associated with trade‐offs between life‐history and dispersal traits.

## Discussion

Our results indicate that both hypotheses about dispersal attenuation behind invasion fronts appear to be at play, but under different circumstances. Spatial sorting operates when there is a persistent density gradient on the invasion front. When we maintained this gradient in the model by allowing the population to grow in a density‐independent manner, the frequency of slow dispersers increased after colonization, even in the absence of natural selection (i.e., no trade‐off between dispersal and fitness; Figs. 1 and 2, upper left). Thus, in the early stages of colonization when population growth is exponential, spatial sorting could be an important mechanism driving dispersal attenuation. In the density‐dependent case, dispersal also attenuated strongly when a life‐history trade‐off operated at high densities and when traits were being observed long after the initial colonization of a location. Under those circumstances, life‐history trade‐offs have plenty of time to operate, the mark of spatial sorting has long vanished (after many generations of gene flow in the absence of a gradient in population density), and a spatially homogenous, slow phenotype will dominate. By contrast, when life‐history trade‐offs only operate at low densities or are absent altogether, the long‐term outcome of dispersal evolution is more complicated. In this case, we observed the spread of fast phenotypes from relatively recently invaded areas back toward the invasion origin and vice versa (Figs. 1 and 2, lower left). This happens because, in the absence of a density gradient or a fitness trade‐off with dispersal propensity, dispersal acts solely as a homogenizing force across the range.

Quantitative details about the long‐term frequencies of dispersal types will depend on additional subtleties, including how quickly equilibrium densities are attained relative to the timescale of spatial spread. Our results show that long‐term, rangewide frequencies of fast and slow types are potentially quite sensitive to the relative dispersal advantage of the fast type and the extent of the spatial domain being invaded. With empirical estimates indicating that spread often proceeds for on the order of 10–100 generations (Perkins 2012) and with mounting evidence of extensive variability in dispersal traits (Hughes et al. 2003; Simmons and Thomas 2004; Leotard et al. 2009; Lindstrom et al. 2013; Lombaert et al. 2014), there is good reason to suspect that spatial sorting should guarantee fast types a lasting advantage over their slow counterparts in many instances of range expansion in nature. After all, we have shown (in the density‐independent case) that, at best, spatial sorting alone can only restore dispersal propensity back to preinvasion levels. Spatial sorting alone cannot account for gains by the slow type beyond preinvasion levels, and only then in the unlikely case of a complete lack of density regulation.

Together, our results suggest that the most likely explanation for empirically observed declines in dispersal following invasion is natural selection due to trade‐offs between dispersal and life‐history traits. In the bluebird example, aspects of that species' natural history consistent with this explanation include that local populations are strongly density‐regulated (by limited nest sites), the process of recolonization by the slow type happens within only a few generations, and there is a strong genetic correlation between dispersal propensity and life‐history traits, particularly at high densities (Duckworth and Badyaev 2007; Duckworth 2008; Duckworth and Kruuk 2009). Aspects of the cane toad's natural history are also consistent with our conclusion about the necessity of trade‐offs for the evolution of dispersal attenuation. Extensive variation in the dispersal propensities of cane toads, a well‐established genetic basis of variation in dispersal and life‐history traits, and a spread phase that has unfolded over vast distances and dozens of generations (Phillips et al. 2008; Alford et al. 2009; Lindstrom et al. 2013) are all conditions that, in the absence of life‐history trade‐offs, appear highly unfavorable for postcolonization dispersal attenuation. Based on our results, the most likely mechanism for the observation of postcolonization dispersal attenuation in cane toads is the presence of trade‐offs between dispersal and fitness. This expectation of a trade‐off is supported by recent observations that highly dispersive invasion‐front toads have a lower reproductive rate than their conspecifics from the range core (Hudson et al. 2015). It is still possible, however, that spatial sorting was an important driver of postcolonization dispersal attenuation in cane toads, and we note that additional empirical observations guided by our theoretical predictions could help resolve this question in the future (Table 2).

**Table 2:** Interpreting empirical observations in light of theoretical results described here

Empirical observation	Theoretical interpretation
Before equilibrium density has been attained at a site postcolonization, modest dispersal attenuation not beyond preinvasion levels	Spatial sorting is definitely operating, and a trade‐off between dispersal propensity and fitness may or may not be operating
Before equilibrium density has been attained at a site postcolonization, rapid dispersal attenuation beyond preinvasion levels	A trade‐off between dispersal propensity and fitness is definitely operating, and spatial sorting is operating to an unknown extent
After equilibrium densities have been attained rangewide, attenuating dispersal beyond preinvasion levels across the range	A trade‐off between dispersal propensity and fitness is definitely operating, and spatial sorting is definitely not operating
After equilibrium densities have been attained rangewide, increasing dispersal near the invasion origin and attenuating dispersal in more recently invaded populations	A trade‐off between dispersal propensity and fitness is not operating. Instead, gene flow is homogenizing dispersal phenotypes across the range

Evaluating the extent to which different conditions apply in recently expanded, spatially distributed species, such as the aforementioned bluebirds and cane toads, suggests a tantalizing possibility that patterns of dispersal evolution following range expansion could be used to infer the existence of trade‐offs between dispersal and life‐history traits. Such trade‐offs are often posited in theoretical studies (Ronce 2007), but empirical evidence of their existence is scarce, coming primarily from flight–fecundity trade‐offs in insects (Hughes et al. 2003; Duthie et al. 2015). A primary reason for this paucity of examples is that it is logistically difficult to measure relevant variables (life‐history and dispersal traits) and then to be sure that all relevant life‐history traits have been taken into account (Ronce 2007; Phillips et al. 2010). Negative relationships between fecundity and dispersal, for example, could be canceled out by negative correlations between fecundity and age to maturity, which would go undetected unless all traits are measured. Thus, observation of postcolonization dispersal attenuation could provide a novel, and very useful, clue about the existence of trade‐offs, even if the proximate traits remain unidentified.

There are, however, a number of limitations that must be kept in mind when reconciling empirical results with those from our theoretical study. One important limitation is that it could take a very long time for the long‐term behavior that we studied to supplant prolonged periods of transient behavior. Ultimately, our model does not allow for stable clines in dispersal propensity, yet clines are clearly manifest across many species' ranges for years after colonization (Cwynar and MacDonald 1987; Simmons and Thomas 2004; Alford et al. 2009; Leotard et al. 2009; Lombaert et al. 2014). Temporal trends in dispersal clines measured at fixed locations across a recently established range should nonetheless yield empirical signatures consistent with one model scenario or another. Another limitation of our model is that it eschews a number of details that could be important in certain systems, including demographic stochasticity, mutation, mating system, relatedness, and Allee effects, all of which have known relevance to dispersal evolution (Cadet et al. 2003; Burton et al. 2010; Travis et al. 2010; Hargreaves and Eckert 2013; Shaw and Kokko 2015). One of the most important reasons to consider such factors in future work is to allow for the possibility of selective forces on dispersal not accounted for by our model (Ronce 2007). For example, dispersal to avoid competing with kin could counteract other forces that select for dispersal attenuation (van Valen 1971). Altogether, such details will be most important in system‐specific models that evaluate the quantitative plausibility of alternative hypotheses about the drivers and consequences of dispersal evolution in a given natural system (e.g., Perkins et al. 2013).

Although system‐specific modeling and observation will be crucial for future studies, our analysis provides an important first suggestion that dispersal propensity typically only shows long‐term attenuation in density‐regulated populations when there is a trade‐off between dispersal and fitness operating at high density. If these trade‐offs are prevalent in nature, then the long‐term implications of dispersal evolution during invasion are likely modest. Gradients in dispersal across the invaded range will, in the absence of alternative fitness peaks, ultimately be transient phenomena, and diversification of life histories driven by spread may be unusual. Thus, the existence of trade‐offs is critical to determine. Importantly, our model suggests that dispersal phenotypes at a location will rarely reflect the phenotypes that first colonized that location, and the course of evolution after colonization can be used to determine whether or not a trade‐off is operating. Although we do not yet know how prevalent trade‐offs between dispersal and life‐history traits are in nature, cuing on patterns of postcolonization dispersal attenuation now gives us a new way of looking for them.

## Conflict of Interest

None declared.
